# Technical Characteristics and Biomedical Applications of Flexible Pressure Sensor Matrices: A Scoping Review

**DOI:** 10.3390/s26061971

**Published:** 2026-03-21

**Authors:** Stefano Cimignolo, Damiano Fruet, Giandomenico Nollo, Michela Masè

**Affiliations:** Department of Industrial Engineering, University of Trento, 38123 Trento, Italy; damiano.fruet@unitn.it (D.F.); giandomenico.nollo@unitn.it (G.N.)

**Keywords:** pressure-mapping systems, resistive and capacitive sensors, wearable interfaces, pressure distribution, sensor sheets, flexible pressure sensor arrays, non-invasive clinical monitoring, plantar pressure, patient monitoring, pressure ulcer prevention

## Abstract

Flexible pressure sensors have been increasingly proposed for clinical monitoring applications. However, the available evidence on the technical characteristics and the biomedical applications of these technologies remains fragmented. To fill this gap, this scoping review aimed to map the available literature (i) to identify the existing flexible pressure sensor matrices proposed for biomedical applications, their technical characteristics, and usage contexts, and (ii) to determine the systems integrated into bed-based support surfaces for clinical monitoring functions. The scoping review was conducted following the guidelines of the Preferred Reporting Items for Systematic Reviews and Meta-Analyses extension for scoping reviews. PubMed, Scopus, and Web of Science databases were systematically searched to identify studies published between 2015 and 2025 that describe flexible pressure sensor matrices for biomedical monitoring and care applications. A total of 5021 records were screened, and 45 studies were included. Existing flexible pressure sensor matrices were mainly based on resistive and capacitive principles. Systems integrated into clinical support surfaces were primarily used for pressure distribution and posture monitoring, and spanned from experimental prototypes to commercially available technologies. A lack of technical specifications and relevant heterogeneity was observed among the studies. Flexible pressure sensors demonstrated potential for clinical monitoring, but standardized technological reporting and clinical validation protocols are needed to develop technically robust and clinically oriented pressure sensing solutions.

## 1. Introduction

Pressure-sensing technologies have been increasingly adopted in clinical contexts for the continuous monitoring of body–support surface interactions and the improvement of patient care, comfort, and safety [1]. These systems play an important role in multiple scenarios. Pressure sensors integrated into medical beds have been used to prevent pressure ulcers or accidental falls [2,3,4,5,6,7,8,9,10,11,12], while sensorized insoles have been utilized in the management of the diabetic foot and the prevention of ulcer formation [13,14]. Indeed, prolonged interface pressures, exceeding ∼30–32 mmHg, have been associated with the risk of tissue ischemia and pressure injury development [15,16,17], while plantar pressures higher than ∼200 kPa are considered critical in the pathogenesis and recurrence of diabetic foot ulcers [13,18].

The integration of these technologies into healthcare facilities, such hospital and nursing homes, has been supported by technological progress leading to the development of thin, flexible, and minimally invasive sensors [19]. These sensors generate a signal, such as a voltage or current, that is proportional to the applied pressure [20]. Pressure transduction to an electrical signal can be obtained based on distinct working principles. The most common sensor types and working principles are described in Section 1.1 and include resistive sensors [21,22], capacitive sensors [23,24], and piezoelectric sensors [25,26]. Depending on the application, pressure sensors can be integrated into different surfaces, such as cushions [10], beds or mattresses [27,28], and shoe insoles [14,29]. Different pressure sensors present distinct structural characteristics (i.e., size, thickness, or geometry) and performance features (i.e., measurement range, spatial resolution, or hysteresis) [30], which may severely impact the overall performance of the system. The optimal sensing technology should be chosen based on technical characteristics and tuned to the specific clinical applications. However, pressure sensor specifications are often incompletely reported, and heterogeneity in the technical data and usage conditions of the sensors are present among studies, which limits the comparison and interpretation of results.

In this context, the aim of this scoping review was to provide a structured overview of pressure-sensing technologies employed for pressure distribution assessment in biomedical and clinical contexts. To achieve this, we mapped and summarized the evidence published over the last 10 years on flexible pressure-sensing technologies based on multi-sensor or sensor array configurations. These technologies were examined in relation to their integration into external and non-invasive support surfaces, including wearable interfaces (e.g., shoe insoles) and fixed platforms (e.g., mattresses and cushions). The review addressed the following specific objectives: (i) to identify existing flexible pressure sensor arrays and describe their technical specifications and biomedical contexts of application; (ii) to describe the pressure monitoring systems integrated into bed-based support surfaces, their proposed clinical application context, and their test population.

### 1.1. Working Principle and Structure of Pressure Sensors

**Figure 1:**
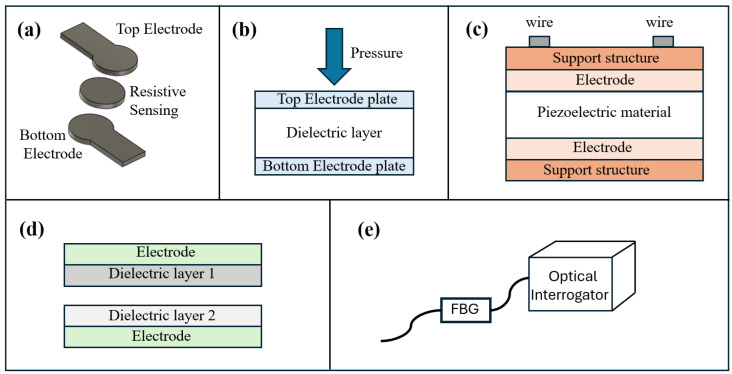
Schematic representation of the main pressure sensing transduction mechanisms used in flexible pressure sensors: (**a**) resistive pressure sensor; (**b**) capacitive pressure sensor; (**c**) piezoelectric pressure sensor; (**d**) triboelectric pressure sensor; and (**e**) optical pressure sensor based on Fiber Bragg Grating (FBG) and optical interrogation.

Flexible pressure sensors are usually classified based on their transduction mechanism and architecture. In biomedical applications, the main transduction technologies include resistive, capacitive, piezoelectric, triboelectric, and optical sensors (Figure 1). Regardless of the transduction principle, the flexibility of the sensor arrays is determined by the structural design and the supporting materials. Sensors are mostly integrated into polymer substrates, characterized by a low elastic modulus and small thickness (e.g., elastomers, silicone, and thermoplastic polyurethane). These materials allow the system to be flexible and to adapt to different surfaces [31,32,33,34].

#### 1.1.1. Resistive Pressure Sensor

Resistive sensors are generally composed of a sensitive layer integrated on a deformable substrate and connected to electrodes for the measurement of the electrical resistance (Figure 1a) [31,35]. The working principle is based on the measurement of the variation in electrical resistance caused by mechanical deformation. When an external pressure is applied, the sensitive layer is compressed or deformed, causing a measurable change in the electrical resistance that is reconducible to the applied pressure [36,37,38].

#### 1.1.2. Capacitive Pressure Sensor

Capacitive pressure sensors are structured as a parallel plate capacitor (Figure 1b). They are composed of a dielectric layer placed between two parallel plates, with electrodes on both sides of the dielectric layer [39]. Usually, one of the plates is fixed, while the other plate is able to move and respond to mechanical pressure [31]. The working principle of these sensors is based on the measurement of the variation in the electrical capacitance in response to pressure. When pressure is applied to the sensor, the distance between the plates and the dielectric properties of the material between them are modified. These variations produce a measurable change in the capacitance that is proportional to the applied pressure.

#### 1.1.3. Piezoelectric Pressure Sensor

Piezoelectric sensors are active sensors, composed of piezoelectric materials, that do not require external energy to produce output signals [40]. These sensors usually consist of a piezoelectric material, a support structure, electrodes, and electrical wires (Figure 1c). The working principle is based on the piezoelectric effect. When a piezoelectric material is subjected to an external pressure, electric charges are generated on both the surfaces of the material, which produces a measurable voltage between the two surfaces [31,41].

#### 1.1.4. Triboelectric Pressure Sensor

Triboelectric pressure sensors are composed of two materials with different tendencies to gain or lose electrons. These materials are placed in contact with each other and connected to electrodes (Figure 1d) [42,43]. The working principle of these sensors is based on the coupling between contact electrification and electrostatic induction. When an external pressure is applied, the two materials come into contact and an electric charge transfer occurs between their surfaces. When the pressure is removed, the separation of the layers creates a potential difference between the electrodes, which induces a measurable flow of charges in the external circuit [31,42].

#### 1.1.5. Optical Pressure Sensor

Optical pressure sensors are usually based on optical fibers, where periodic modulations of the refractive index, called Bragg gratings, are created. The optical fiber is integrated into a mechanical structure that transfers the applied load to the sensor and that is connected to an external optical interrogation system for the analysis of the reflected light (Figure 1e) [44]. The working principle is based on the variation in the Bragg wavelength caused by mechanical deformation. When pressure is applied, the structure is deformed and the grating periodicity is altered, affecting the effective refractive index. This produces a shift in the reflected wavelength, whose magnitude is proportional to the deformation and, therefore, to the applied pressure [44,45].

## 2. Materials and Methods

The scoping review was conducted following the guidelines of the Preferred Reporting Items for Systematic Reviews and Meta-Analyses (PRISMA) extension for scoping reviews (PRISMA-ScR) [46]. The completed PRISMA-ScR checklist is provided in the Supplementary Materials.

### 2.1. Research Questions

In line with the objectives of this scoping review, two research questions (RQs) were formulated:

$RQ_{1}.$ What types of flexible pressure sensor arrays are integrated into external, non-invasive support surfaces for pressure distribution assessment in biomedical and clinical settings, and what are their technical characteristics?
$RQ_{2}.$ Among the identified flexible pressure-sensing systems, which ones are applied in bed-based settings, what are the proposed clinical contexts of use, and what are the study populations used for device testing?

### 2.2. Eligibility Criteria

The literature search was performed by three authors (SC, MM, and DF) to identify studies published in the last 10 years that used pressure-sensing and pressure-mapping technologies to assess pressure distribution in biomedical and clinical settings. The search strategy design is schematized by the inclusion criteria in Table 1, categorized according to the broad Population–Concept–Context (PCC) mnemonic recommended for scoping reviews [47,48]. This scoping review included only studies that developed or applied pressure-sensing and pressure-mapping technologies for biomedical and healthcare settings. In particular, it focused only on multi-sensor or sensor array configurations, enabling spatial pressure distribution assessment. These systems had to be integrated into external, non-invasive support surfaces, such as mattresses, cushions, sheets, or insoles. The exclusion criteria were as follows: studies with devices not intended for biomedical applications; studies with single sensor setups providing only local measurements; and studies with sensors directly attached to the skin or body segments. The search was restricted to articles published in English in peer-reviewed journals. No restrictions on studies’ designs were posed. Abstract presentations, proceedings, and reviews were excluded.

**Table 1:** Inclusion criteria for the scoping review summarized according to the Population–Concept–Context (PCC) mnemonic, recommended for scoping reviews [47,48].

Population	Concept	Context
Healthy volunteers and clinical patients of any sex and age.	Multi-sensor pressure arrays and pressure-mapping technologies for pressure distribution assessment, integrated into external, non-invasive support surfaces.	Biomedical and clinical application settings.

### 2.3. Information Sources, Search Strategy, and Study Selection

A systematic search was performed in Scopus, PubMed, and Web of Science electronic databases to identify primary references from January 2015 to October 2025. In accordance with the PCC, the search string was organized into three conceptual blocks. Within each block, the search terms were combined using the Boolean operator OR, while the three blocks were connected using the Boolean operator AND. The search terms associated with each block are reported in Table 2. The specific strings used for each electronic database are reported in Table S1 in the Supplementary Materials. The search strategy was designed to be sufficiently broad to encompass the diverse terminology and types of pressure monitoring sensors. Studies employing body-mounted or skin-attached sensors were subsequently excluded during the screening phase, in accordance with the predefined eligibility criteria.

**Table 2:** Search terms used in the three blocks for the construction of the search string.

Technology Terms	Monitoring Terms	Clinical Context Terms
“pressure mapping”; “pressure distribution”; “interface pressure”; “pressure sensing”; “pressure sensing array”; “pressure mat”; “pressure sensing pad”; “support surface”; “smart mattress”; “force sensing resistor”; FSR; “wearable pressure sensor”; “piezoresistive”; “piezoelectric”; “triboelectric”; “capacitive”; “flexible pressure sensor”; “tactile sensor”; “pressure imaging”	“pressure monitoring”; “continuous monitoring”; “dynamic pressure”; ”pressure assessment“; ”real-time monitoring“; ”long-term monitoring”; ”remote monitoring”; “body position monitoring”; “mobility monitoring”; “continuous data acquisition”; “sensor network”; “IoT”; “wearable”	patient *; clinical; hospital; rehabilitation; “acute care”; ICU; “intensive care”; “long-term care”; LTC; geriatric *; immobil *; bedridden; “bed ridden”; “pressure injury prevention”; “pressure ulcer”; “bedsore”; “bed sore”; “nursing care”; “nurse led clinic *”; motion *; reposition *; “turning”

* indicates truncation used in the search strategy to include multiple word endings.

All the retrieved records were imported into Rayyan online platform [49] for duplicate removal, screening, and studies’ selection. According to the PRISMA-ScR guidelines [46], study selection was conducted in two stages by two reviewers (SC and MM). First, the titles and abstracts were screened to assess their relevance and compliance with the predefined eligibility criteria. Second, the full texts of the potentially relevant records were examined to confirm their suitability for inclusion in the scoping review.

### 2.4. Data Extraction and Synthesis

Two reviewers (SC and DF) independently extracted the technical, experimental, and demographic data from the selected studies. When disagreement occurred, they reviewed the papers together with a third reviewer (MM) to reach a joint conclusion. The data extraction was guided by the RQs. For $RQ_{1}$, the following relevant information was extracted: the application type, device name, transduction modality, structural characteristics (size and thickness), pressure range, array configuration (number of sensors and spatial resolution), and support material. These characteristics were summarized using frequency distributions and comparative tables. Performance parameters, such as accuracy and hysteresis, were also screened during data extraction, but they were not included in the tables due to inconsistent reporting. For $RQ_{2}$, the following information was extracted: the study setting, clinical context, number of participants, and the demographic and anthropometric characteristics of the participants (age, height, weight, and body mass index (BMI)). This information was summarized in a comparative table.

### 2.5. Bias Assessment

Consistent with the objective of scoping reviews, which is to provide a thorough and broad survey of the literature, we did not perform a formal bias assessment.

### 2.6. Statistical Analysis

A meta-analysis was not performed, given the heterogeneity in study designs and outcomes among the selected studies.

## 3. Results

### 3.1. Study Selection

**Figure 2:**
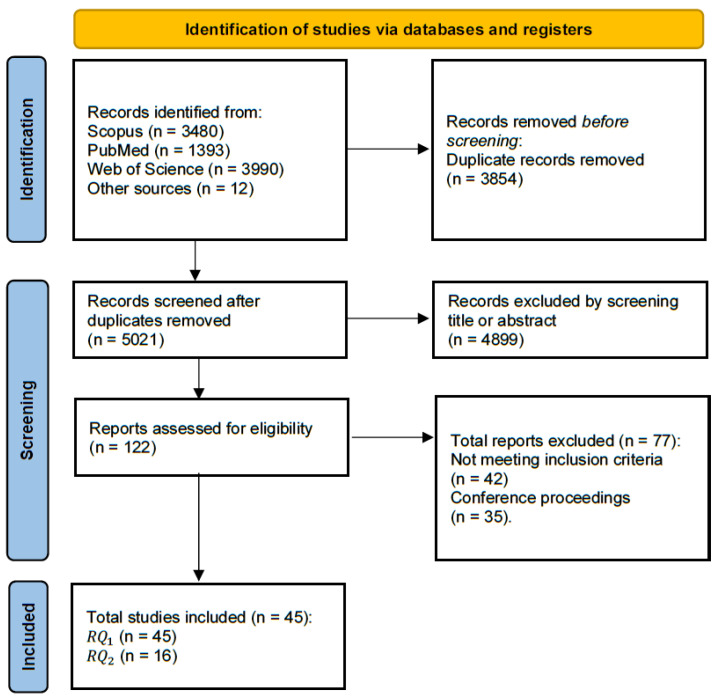
Selection process for the studies included in the scoping review. The Preferred Reporting Items for Systematic Reviews and Meta-Analyses [PRISMA-ScR [46]] flow diagram depicts the number of records identified, included and excluded, and the reasons for exclusion, through the different phases of the scoping review.

Figure 2 summarizes the entire process of the systematic literature search, study selection, and classification. As can be seen, 5021 records were identified after duplicate removal. Of these, 4899 references were excluded after reading the titles and abstracts, and 122 were retrieved for further evaluation. Of these, 77 studies were excluded because they did not fulfil the inclusion criteria. In total, 45 studies were included in the qualitative synthesis on pressure sensors ($RQ_{1}$). These studies are described in Section 3.2.

A subgroup of 16 studies analyzed pressure-sensing systems integrated into bed support surfaces for clinical purposes and were included in the sub-synthesis $RQ_{2}$. These studies are described in Section 3.3.

### 3.2. Technical Characteristics of Pressure-Sensing Technologies

**Figure 3:**
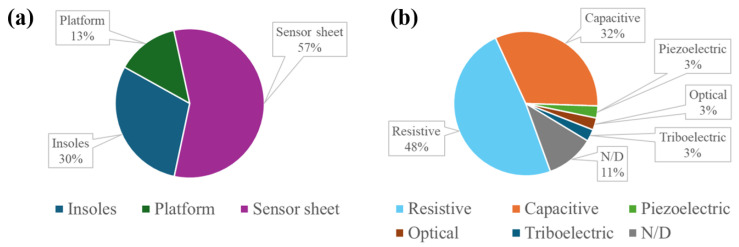
Distribution of pressure-sensing systems by support surface category (**a**) and transduction modality (**b**); N/D indicates not declared.

The systematic literature search yielded 45 studies that described flexible pressure sensor arrays integrated into external surfaces in biomedical and clinical settings. The technical characteristics and application settings of these sensing systems are summarized in Table 3, Table 4 and Table 5. As shown in Figure 3a, approximately 57% of pressure detection systems were integrated into sensor sheets, 30% into insoles, and 13% into platform-type systems. In terms of tranduction mechanisms, 48% of the identified systems were based on resistive sensors and 32% on capacitive ones, while piezoelectric, optical, and triboelectric solutions were less common (Figure 3b).

**Figure 4:**
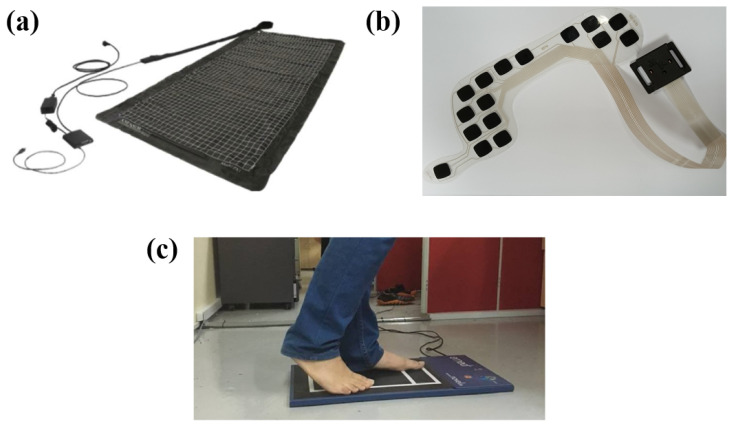
Representative examples of pressure-sensing systems integrated into different support surfaces for pressure distribution assessment in biomedical applications. (**a**) Flexible pressure-sensing sheet used for monitoring body–surface interactions (modified from [78]); (**b**) insole for plantar pressure monitoring; and (**c**) pressure measurement platform (modified from [79]).

The identified sensor sheet systems are described in Table 3 and an illustrative device is displayed in Figure 4a. Sensors sheets were designed to be placed between the body and the support surface. Support surfaces included beds and chairs (e.g., wheelchairs). The size of the sheet depended on the body area to be monitored, ranging from 67 mm× 138 mm for a chair setup [50] to 1950 mm × 863 mm for bed setups [51]. The thickness was generally limited to a few millimeters [52]. The number of sensors largely varied among devices, ranging from a few elements [9,53] to high-density arrays with up to 6136 sensors [54,55,56]. The most common working principles were resistive [52,57,58] and capacitive [27,59,60]. The pressure range for sensor sheet systems varied among devices, but several systems reported an upper limit of approximately 34 kPa (255 mmHg) [28,52,61]. Common supporting materials included synthetic rubber, polyimide, polyester films, and polyethylene terephthalate (PET). These materials conferred flexibility to pressure-sensing systems, enabling them to conform to different support surfaces while maintaining mechanical flexibility [50,51,52,62,63].

The characteristics of the identified insole systems are reported in Table 4 and an illustrative example is shown in Figure 4b. Insole systems are designed to be wearable and fit the plantar surface. Thus, as reported in [29], their size changed with shoe size, and their thickness was a few millimeters [64,65,66]. The number of sensors ranged from a few sensors [33,45,65,67,68,69,70,71], placed in specific plantar areas (e.g., the heel and metatarsal region), to high-density sensor matrices, which could integrate up to 99 sensors per foot [14,29,67,72,73,74]. The most common sensing technologies were resistive [33,68,71,72], capacitive [65,69,72,73], and piezoelectric sensors [75], while optical sensors, such Fiber Bragg Grating, were less common [45].

The supporting materials included polymeric substrates (e.g., PET) [4,72], silicone-based elastomers (e.g., Ecoflex or room-temperature vulcanizing (RTV) silicone) [68,71], thermoplastic polyurethane (TPU) [75,76], foam-based substrates (e.g., ethylene–vinyl acetate (EVA)) [33], and conductive textiles [65]. In optical sensing systems, polymer optical fibers, based on cyclic transparent optical polymers (CYTOPs), were used to implement Fiber Bragg Grating sensors [45]. These materials facilitated the system’s flexibility and its mechanical compliance with the plantar surface.

The characteristics of the platform-type systems are summarized in Table 5 and an illustrative case is presented in Figure 4c. Platform-type systems were constituted by large surfaces [43,72,77], which enabled pressure data collection from one foot or both feet simultaneously. The large surface could include many sensing elements—up to 10,000 sensors in [77]. The most used sensing technologies were resistive and capacitive sensors [72], while triboelectric nanogenerator-based (TENG-based) sensors remained limited to experimental setups [43].

### 3.3. Pressure-Sensing Technologies Embedded in Bed-Based Support Surfaces

Among the sensor sheet systems in Table 3, we identified a subgroup of 16 studies, which described pressure-sensing systems integrated into bed support surfaces for clinical use ($RQ_{2}$). Table 6 summarizes the settings of use, the clinical contexts, and the study populations on which the devices were tested. The majority of the studies were carried out in controlled settings or hospital laboratories [51,56,58,62,80,81], while only five studies were performed in real-life settings, such as nursing homes [27] or operating rooms [50,82]. Seven studies included only healthy volunteers and were mostly aimed at evaluating comfort or classifying body postures [80,83]. Seven studies focused on vulnerable groups, such as hospitalized patients at risk of pressure injuries [50] and pediatric populations undergoing neuro-development assessment [58,60]. The number of participants was generally limited, ranging from small groups of 11–12 participants [51,80] to larger groups of 116 subjects [62]. Anthropometric data, such as BMI, were usually reported by studies on healthy adults, while they were absent or incomplete in clinical studies [2,54,82].

The most common clinical contexts of use included pressure injury prevention [54,78], posture/mobility classification [57], and infant neuromotor assessment [58,60]. In the context of pressure injury prevention, a study conducted in nursing homes applied sensor sheets to quantitatively compare the comfort of spring-based surfaces versus conventional systems, but the differences between the two systems did not reach statistical significance [78]. The performance of continuous pressure monitoring in residents with pressure injuries revealed high inter-subject variability in the duration of static postures, which ranged between 1.7 to 19.8 h, and a high number of patients (17 over 22), who spent at least 60% of the time in static postures longer than 2 h [27]. In intraoperative settings, pressure monitoring was used to evaluate intraoperative protocols based on pressure map-guided repositioning [82], as well as to investigate the factors associated with the occurrence of pressure injury [50]. In particular, an extreme BMI and a prolonged contact with the surface (>2 h) were the main factors associated with pressure injury, supporting the clinical relevance of pressure-mapping systems. In the context of posture and in-bed mobility classification, a biomechanical analysis based on pressure-mapping signals indicated the contact area as the most informative variable, with 7 of 12 signals achieving an area under the curve>0.5 [80]. The analysis of pressure data with machine learning techniques showed a capability to detect static postures and transitions with a classification accuracy of 82–100% for Naive Bayes, 70–98% for K-Nearest Neighbors, and 69–100% for Support Vector Machine [81]. Pressure monitoring in pediatric populations revealed specific movement patterns related to biomechanical constraints that were only partially overlapped with those of adults [58]. The combination of pressure monitoring with a Convolutional Neural Network showed the capability of accurate movement classification, reaching an accuracy of 81.4% [60].

**Table 3:** Technical characteristics of flexible pressure sensor sheets.

Device Name	Transduction Modality	Size [mm × mm]; Thickness [mm]	Pressure Range [kPa]	No. of Sensors; Spatial Resolution [sensors/cm^2^]	Support Material	References
Conformat Tekscan, Inc., (Boston, MA, USA)	Resistive	471.4 × 471.4; 0.134	0–34	1024; 0.5	N/D	[52,60,61,84]
Cognito Health (Boulder, CO, USA)	Resistive	1506 × 715; 0.1	0–33	240; N/D	N/D	[52]
Model 5330 Tekscan, Inc., (Boston, MA, USA)	Resistive	571.5 × 627.4; 0.762	0–34	1024; 0.5	N/D	[28]
Sensomatt (Branco, Portugal)	Resistive	1850 × 750; N/D	60–135	72; N/D	Synthetic rubber	[62,63]
BIGMAT 2000 v. 5.87 (Nitta Co., Osaka, Japan)	Resistive	440 × 440; 0.4	N/D	2112; 1	N/D	[58]
Customized	Resistive	N/D; N/D	N/D	45; N/D	N/D	[53]
Tactilus (Sensor Product Inc., Madison, NJ, USA)	Resistive	1800 × 780; 0.3	0–13.3	1728; N/D	Polyimide/ Polyester films	[62]
Customized	Resistive	1850 × 950; N/D	N/D	220; N/D	N/D	[57]
Customized	Resistive	1250 × 740; N/D	N/D	195; N/D	N/D	[56]
Tactilus (Sensor Product Inc., Madison, NJ, USA)	Resistive	1988 × 898; 2.5	0–13.8	1728; N/D	Polyimide/ Polyester films	[51]
ForeSitePT, Xsensor, (Calgary, AB, Canada)	Capacitive	762 × 1800; 0.1	0–34.2	5664; N/D	N/D	[27,52,59,80,81]
ForeSitePT, Xsensor (Calgary, AB, Canada)	Capacitive	762 × 1880; N/D	0–34.2	6136; N/D	N/D	[54,55,56]
ForeSiteSS Xsensor (Calgary, AB, Canada)	Capacitive	457 × 457; 0.1	0–34.2	1296; N/D	N/D	[52,55]
ForeSiteIS Xsensor (Calgary, AB, Canada)	Capacitive	1880 × 762; 0.1	0–26.7	1440; N/D	N/D	[52]
ForeSiteOR Xsensor (Calgary, AB, Canada)	Capacitive	N/D; N/D	N/D	N/D; N/D	N/D	[82]
Xsensor X3 (Calgary, AB, Canada)	Capacitive	813 × 2032; N/D	0–26.7	1024; N/D	N/D	[78,85]
ePad-ExtraS50, eBio Tech., (New Taipei, Taiwan)	Capacitive	670 × 1380; N/D	N/D	252; N/D	PET	[50]
SR Soft Vision (Sumitomo Riko, Aichi, Japan)	Capacitive	355 × 355; 0.2	0–26.7	256; N/D	Smart rubber	[52]
Robotic mattress (LEIOS, Molten Corporation, Hiroshima, Japan)	N/D	N/D; N/D	0–13.3	1281; N/D	N/D	[83]
Vista Medical FSA SoftFlex 2048 (Vista Medical Ltd., Winnipeg, MB, Canada)	N/D	N/D; N/D	0–133.3	2048; N/D	N/D	[2]
Customized	N/D	N/D; 0.4	N/D	28; N/D	N/D	[9]

(N/D: not declared).

**Table 4:** Technical characteristics of plantar pressure insoles.

Device Name	Transduction Modality	Insole Size [EU]; Thickness [mm]	Pressure Range [kPa]	No. of Sensors per Insole; Spatial Resolution [Sensors/cm^2^]	Support Material	References
SurroSense Rx Orpyx Medical Technologies Inc. (Calgary, AB, Canada)	Resistive	N/D N/D	N/D	4; N/D	N/D	[67]
F-Scan, Tekscan (Boston, MA, USA)	Resistive	N/D; <1	N/D	N/D; 3.9	PET	[4,64,66,72]
Customized	Resistive	N/D; 2.5 mm	N/D	8; N/D	Ecoflex	[68]
Customized	Resistive	42; N/D	N/D	11; N/D	EVA foam	[33]
Customized	Resistive	43; N/D	N/D	5; N/D	RTV Silicone rubber	[71]
Pedar-X Novel GmbH, (Munich, Germany)	Capacitive	22–49; N/D	15–600 or 30–1200	99; N/D	N/D	[14,29,67,72,73,74]
FeetMe (Paris, France)	Capacitive	37–42; N/D	N/D	18; N/D	N/D	[69]
Customized	Capacitive	N/D; 1.7	0–110	10; N/D	Conductive textile	[65]
Fabricated by ITRI (Hsinchu County, Taiwan)	Piezoelectric	N/D N/D	0–965	89; N/D	TPU	[75,76]
Customized	Optical	N/D; 10	10–1500	5; N/D	CYTOP	[45]
Customized	N/D	N/D; N/D	≤300	27; N/D	Velostat	[70]

(N/D: not declared).

**Table 5:** Technical characteristics of pressure measurement platforms.

Device Name	Transduction Modality	Size [mm × mm]; Thickness [mm]	Pressure Range [kPa]	No. of Sensors; Spatial Resolution [Sensors/cm^2^]	Support Material	References
Walkway HRV5 (Tekscan, Norwood, MA, USA)	Resistive	2438 × 447; N/D	0–862	N/D; 4	N/D	[72]
Footscan Entry Level (RSSCAN, Ipswich, England)	Resistive	976 × 325; 12	0–1270	8192; 2.5	N/D	[72]
freeMed, Sensor Medica (Guidonia Montecelio, IT, USA)	Resistive	1200 × 500; 8	0–1500	10,000 N/D	N/D	[77]
Emed-X (Novel, Pittsburgh, PA, USA)	Capacitive	476 × 320; N/D	N/D	6080; 4	N/D	[72]
Customized	Triboelectric	N/D; N/D	N/D	64; N/D	Silicone	[43]

(N/D: not declared)

**Table 6:** Clinical contexts of application and study populations of the studies on flexible pressure-sensing systems integrated into bed-based support surfaces.

Study	Setting	Context	Population Category	No. of Participants (Sex)	Age [Years]	Height [m]	Weight [kg]	BMI [kg/m^2^]
Caggiari et al. [80]	Hospital laboratory	Postural changes when lying	Healthy volunteers	11 (6M, 5F)	32	1.72 ± 0.51	71.2 ± 25.2	[20.4–30.1]
Caggiari et al. [81]	Hospital laboratory	Posture detection and pressure ulcers’ prevention	Healthy volunteers	19 (9M, 10F)	32 (training group), 34 (test group)	1.70 (training group), 1.73 (test group)	72.0 (training group), 68.9 (test group)	[19–30] (training group), [19–28] (test group)
Caggiari et al. [27]	Private, residential and nursing homes	Pressure ulcer and posture/ mobility monitoring	Patients with pressure ulcers	22	[32–95]	N/D	N/D	[17.5–47]
Fryer et al. [55]	Specialist spinal cord injury center	Pressure ulcer assessment and movement monitoring	Inpatient spinal cord injury patients	12	[18–77]	N/D	N/D	[21–30]
Hajari et al. [2]	N/D	Pressure injury	N/D	13	N/D	N/D	N/D	N/D
Laurino et al. [56]	University laboratory	Sleep monitoring and sleep quality assessment	Healthy volunteers	34 (21M, 13F)	47.9	N/D	N/D	N/D
Saegusa et al. [83]	N/D	Comfort	Healthy volunteers	20 (7M, 13F)	32.4 ± 8.3	1.62 ± 0.77	N/D	Reported as categories only
Fonseca et al. [62]	University laboratory	Position classification, sleep disorders and bedded or lying people	Healthy volunteers	60 (36M, 24F)	N/D	[1.45–1.95]	[45–127]	N/D
Beime et al. [78]	Nursing home	Pressure ulcer prevention and mobility assessment during sleep	Elderly nursing home residents	27 (5M, 22F)	81.7 ± 9.5	1.6 ± 0.1	71.3 ± 17.3	26.5 ± 5.5
Kau et al. [57]	University laboratory and Home environment	Sleep posture recognition and sleep quality assessment	Healthy volunteers	Laboratory 18 (15M, 3F) Home 2 (1M, 1F)	Laboratory [22–29] Home [29–73]	Laboratory [1.51–1.83] Home [1.50–1.73]	Laboratory [45–92] Home [46–70]	Laboratory [16.98–28.71] Home [20.04–23.4]
Kobayashi et al. [58]	University laboratory	Motor development and rolling movement analysis	Infants	32 (10M, 22F)	[270–299] days	N/D	[6.7–10.48]	N/D
Kulvicius et al. [60]	University hospital laboratory	Infant neuromotor assessment and early detection of neuromotor disorders	Infants	51 (25M, 26F)	[4–16] weeks of post-term age	N/D	N/D	N/D
Matar et al. [51]	University laboratory	Posture classification for pressure ulcer prevention	Healthy volunteers	12 (10M, 2F)	27.35 ± 5.39	[1.55–1.85]	[50–130]	N/D
Shih et al. [50]	Hospital operating room	Pressure injury	Patients with and without pressure ulcer	47 (32M, 15F)	53.3 ± 11.4	1.63 ± 0.13	66.7 ± 13.2	24.9 ± 4.1
Sving et al. [82]	Hospital operating room	Pressure ulcer prevention	Patients	116 (31M, 85F)	60.9	N/D	N/D	Reported as categories only
Wong et al. [54]	Hospital inpatient units	Pressure ulcer prevention	Patients	678 planned	N/D	N/D	N/D	N/D

Data are reported as the mean and mean ± SD or [range]; M: Male; F: Female; and N/D: Not Declared.

## 4. Discussion

The purpose of this study was to map the literature on flexible pressure sensor arrays integrated into external support surfaces for applications in biomedical and clinical settings. Forty-five studies were selected, which testified a growing research interest on pressure-sensing technologies for clinical pressure monitoring. However, even if many technological solutions existed, the literature mapping showed gaps in the description of their technical characteristics, as well as limitations in their actual transfer to the clinical setting.

The included studies describe pressure-sensing systems integrated in three types of interfaces (Figure 3a): (i) sensor sheets (Figure 4a), (ii) insoles (Figure 4b), and (iii) platforms (Figure 4c). Each interface type has different design features. Sensor sheets are designed to cover wide areas and to ensure flexibility and integration with support materials (i.e., mattresses and cushions), enabling pressure distribution monitoring in bedridden patients or in patients with reduced mobility [50,52,54,78]. The main advantage of these systems is the possibility of continuous, non-invasive, and long-term monitoring without interfering with patients’ comfort. However, the presence of a layer between the sensor sheet and the patient’s body has been shown to affect the transmission of pressure and reduce the ability to detect pressure peaks [63]. In addition, these systems often display lower spatial resolution compared to other configurations, due to the larger covered surface.

Insoles are used to measure plantar pressure [29,69,74]. The main clinical application of these devices is related to the prevention or monitoring of diabetic foot ulcers. The integration of sensors inside the insoles introduces important design constraints. The device has to be thin, flexible, and resistant to the mechanical stress associated with walking. In addition, factors, such as patient comfort, device durability, and adherence to use, could influence the effectiveness of long-term monitoring.

Platforms do not display these limitations, and can have larger surfaces and integrate a higher number of sensors [72,77]. These systems are used in clinical or laboratory environments to analyze pressure distributions during motor activities, such as standing or walking [72,77]. The main advantage of these systems is related the high number of sensors and large surfaces, which allow for the high-resolution mapping of pressure distribution. However, these platforms can only be used in controlled static environments, which limits the possibility of monitoring patients during daily activities.

In terms of the used sensing technologies, our review showed that resistive and capacitive systems were prevalent (nearly 80% of these devices), while the use of piezoelectric, optical, and triboelectric solutions was sparse (Figure 3b). This prevalence suggests a preference for technologies that offer a favorable trade-off of cost, performance, and ease of integration into sensor array systems embedded into external surfaces.

The technical characteristics of the main commercial technologies and prototypes provided in this review (e.g., the number of sensors and overall dimensions) constitute a useful reference for the development of customized systems, enabling designers to build configurations that have been already explored and validated. Despite the growing number of identified technological solutions, the literature mapping highlighted several critical issues in how pressure-sensing systems have been described and evaluated. A first relevant issue emerging from Table 3, Table 4 and Table 5 is that key technical parameters, such as thickness, pressure range, and spatial resolution, were often not reported [2,66,82]. This lack of information limits the interpretation and comparability of the results among studies. For instance, the spatial resolution of the systems is a key parameter to ensure the reliable detection of localized pressure peaks, which are useful clinical outcomes for monitoring the diabetic foot [67]. Similarly, information on the materials used for the supporting substrates was often absent [2,52,72], although these materials can influence mechanical compliance, pressure transmission, and the long-term durability of the sensing systems. A common lack of information among studies concerned performance parameters, such as accuracy, hysteresis, and repeatability (which indeed are not included in Table 3, Table 4 and Table 5). This limitation hindered the possibility of a direct comparison between different measurement systems. Indeed, without these parameters, it is difficult to determine whether the observed pressure variations are caused by real physiological changes or by artefactual intrinsic sensor effects, such as delays or hysteresis of the material. These aspects are particularly important in clinical applications, where the device should be able to distinguish between normal physiological conditions and pathological risk thresholds.

An additional common issue concerned the absence of a rational supporting the design choices. In the majority of studies, the choice of the sensing principle, sensor density, and array configuration was not explained nor related to the clinical scenario of application. This makes it hard to understand whether the choice was driven by clinical needs, engineering constraints, or simply by device availability. Similarly, the studies lacked of a clear link between the technical specifications of the sensors and the clinical meaning of the measurements. For instance, in the monitoring of the diabetic foot, the reduction of the plantar pressure to values below 200 kPa was shown to be related to effective ulcer treatment and prevention of ulcer recurrence [13,18]. Some studies included in this review reported measurement ranges up to 600–1500 kPa [45,67,74], which is far beyond the pressure range of clinical interest. In the context of pressure injuries, pressures higher than 30–32 mmHg, maintained for a prolonged time (>2 h), have been associated with the risk of tissue ischemia [15,16,17]. The bed-integrated sheet sensors described in this scoping review generally covered this range, with the measurement values reaching up to 34 kPa (about 255 mmHg) [55,81]. In several studies, these systems were mainly used to construct pressure maps or to classify posture with machine learning and deep learning algorithms [2,83], but it was not explained how the measured values could support practical clinical decisions, for example deciding the time period after which a patient should be repositioned to prevent a pressure ulcer.

The analysis of the subgroup of studies that integrated pressure-sensing systems into bed-based support surfaces (Table 6) suggests a growing clinical interest for non-invasive monitoring solutions based on sensor sheets. Some studies provided a proof of concept of the actual implementability of these sensors in clinical contexts, while other studies at least suggested their potential applications in real-world clinical environments, such as hospitals, operating rooms, and nursing homes [50,54,82]. However, these studies showed high variability in terms of sensor configurations, use settings, and included populations. Almost half of the studies tested the proposed systems only on healthy volunteers. Instead, only a small number of studies with generally small sample size (groups of approximately 11–12 participants [55,80]) applied the devices in clinically relevant scenarios, such as pressure injury prevention or posture classification [27,57]. In addition, anthropometric information (BMI, age, and weight) regarding the participants was often reported incompletely, especially in studies on vulnerable clinical populations [50,82], which poses generalizability restrictions for use in other populations. Finally, the outcomes considered varied widely among studies and were rarely validated against clinical reference standards.

### 4.1. Limitations of This Scoping Review

Our scoping review has some limitations. The search was limited to peer-reviewed publications, so technical reports and industrial documentation were excluded. This choice may have excluded solutions that are already mature in practice, but are not yet described in the scientific literature.

In addition, we considered only body pressure-sensing systems integrated into external, non-invasive support surfaces, excluding sensors attached to the skin or implantable systems. Although this choice matched the study objectives, it limited the coverage of other device categories that could also play a role in clinical practice.

### 4.2. Directions for Future Development

In line with the objective of this scoping review, the collected evidence suggests some directions for future research. Technical information on sensor characteristics was often lacking in the analysed literature, limiting the comparison between different technologies and suggesting the need for a systematic reporting of technical characteristics in future studies. In detail, to improve reproducibility and clinical translation, future studies should report the following information: (i) sensor thickness, and the substrate material with mechanical properties; (ii) the measurement range; (iii) accuracy, repeatability, sensitivity, linearity, and drift; and (iv) the spatial resolution and sampling frequency.

Finally, many bed-based applications were tested only on small groups and often on healthy volunteers, which do not fully reflect the characteristics of the clinical populations of interest. This limits the extrapolation of the reported results to real-world clinical settings and pathologic populations, where patients often present reduced mobility, extreme BMI variations, or altered tissue conditions that may significantly affect pressure distributions. Future studies should enrol more representative populations, reflecting the clinical scenarios of interest, such as patients with reduced mobility, neuropathy, vascular impairment, or a high BMI. In addition, the main demographic and anthropometric variables of the participants, such as age, BMI, mobility status, comorbidities, and their history of pressure ulcers, should be systematically reported to allow for a better interpretation and comparison of the studies’ results.

## 5. Conclusions

This scoping review systematically mapped the literature on surface-integrated flexible pressure sensor arrays for pressure distribution assessment in biomedical and clinical settings. The identified studies testified a growing variety of technological solutions and a wider range of proposed applications, from wearable devices to systems integrated into bed support surfaces. The systematic summary of the technical information of existent pressure sensor systems constitutes a significant reference framework for the design of future devices. However, our work revealed also limitations in technical reporting, design justification, and the linking between measurements and clinical meaning among studies. Standardized technology reporting and clinical validation protocols are needed in future studies to develop technically robust and clinically oriented pressure sensing solutions.

## Data Availability

All relevant data are reported in the study.

## Supplementary Materials

The following supporting information can be downloaded at https://www.mdpi.com/article/10.3390/s26061971/s1, PRISMA-ScR Checklist; Table S1: Search strategies employed with the three analyzed electronic databases: Scopus, PubMed, and Web of Science.
